# 9-[(*Z*)-2-(4,4,5,5-Tetra­methyl-1,3,2-dioxaborolan-2-yl)ethen­yl]-9*H*-carbazole

**DOI:** 10.1107/S2414314621001425

**Published:** 2021-02-12

**Authors:** Mayu Kanagawa, Kazuto Akagi, Tsunehisa Okuno

**Affiliations:** a Department of Systems Engineering, Wakayama University, Sakaedani, Wakayama, 640-8510, Japan; Sunway University, Malaysia

**Keywords:** crystal structure, 1,3,2-dioxaborolane, conformation

## Abstract

The title compound, C_20_H_22_BNO_2_, has a polarized π-system due to resonance between N—C(H)=C(H)—B and ionic N^+^=C(H)—C(H)=B^−^ canonical structures. The dihedral angles between the ethenyl plane (r.m.s. deviation for C_2_H_2_ = 0.0333 Å) with the ethenyl-*C*(NC_2_-pyrrole) plane (r.m.s. deviation CNC_2_ 0.0423 Å) and the ethenyl-C(BO_2_-1,3,2-dioxaborolane) plane (r.m.s. deviation BCO_2_ 0.0082 Å) are 45.86 (8) and 37.47 (8)°, respectively, and are greater than those found for the previously reported *E*-isomer [Hatayama & Okuno (2012 ▸) *Acta Cryst.* E**68**, o84]. In comparison with the *E*-isomer, the reduced planarity of *Z*-isomer results in a decrease of the contribution of the N^+^=C(H)—C(H)=B^−^ canonical structure.

## Abstract 2

The title compound has a polarized π-system due to resonance between N—C(H)=C(H)—B and ionic N^+^=C(H)—C(H)=B^−^ canonical structures. In comparison with the previously reported *E*-isomer, the reduced planarity of *Z*-isomer results in a decrease of the contribution of the N^+^=C(H)—C(H)=B^−^ canonical structure.

## Graphical abstract

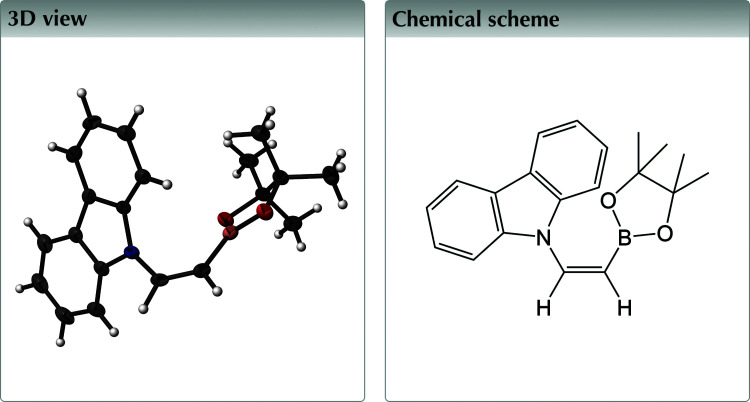

## Structure description

**Figure 1:**
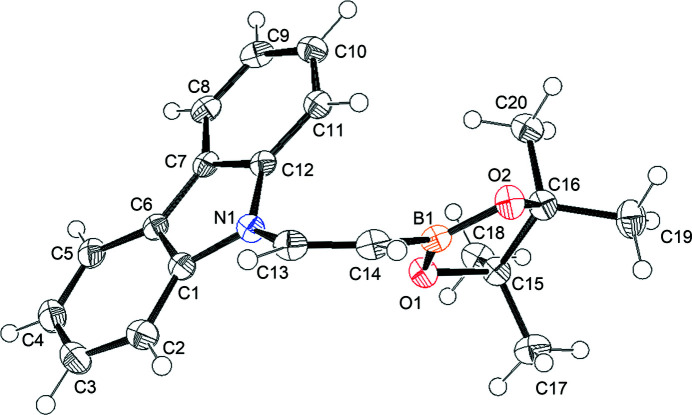
The mol­ecular structure of the title compound showing the atom-numbering scheme with displacement ellipsoids drawn at the 50% probability level and H atoms shown as arbitrary spheres.

The title compound, C_20_H_22_BNO_2_, has a hybrid π-conjugated system comprising an N—C(H)=C(H)—B unit (Fig. 1 ▸). The insertion of a π-conjugated system in the N—B bond can give a highly polarized π-system as a result of the contribution of an ionic canonical structure, N^+^=C(H)—C(H)=B^−^. However, the contribution of the ionic canonical structure is very small when *p*-phenyl­ene is inserted into the N—B bond (Yuan *et al.*, 2006 ▸). By contrast, there is a significant contribution of the ionic canonical structure when a C≡C bond is inserted into the N—B bond (Onuma *et al.*, 2015 ▸). The crystal structure of one isomer of the C=C bond-inserted system, namely 9-[(*E*)-2-(4,4,5,5-tetra­methyl-1,3,2-dioxaborolan-2-yl)ethen­yl]-9*H*-carbazole has been reported (Hatayama & Okuno, 2012 ▸), which is an *E*-isomer of the title compound. In this work, the preparation of the *Z*-isomer is reported as is a comparison of the crystal structures of the isomers.

The dihedral angles between the C13/C14/H13/H14 plane (r.m.s. deviation 0.0333 Å) and the N1/C1/C12/C13 plane (r.m.s. deviation 0.0423 Å) and B1/O1/O2/C14 plane (r.m.s. deviation 0.0082 Å) are 45.86 (8) and 37.47 (8)°, respectively. The relatively large angles result in steric repulsion between carbazolyl and Bpin (pin = pinacolato) residues. The equivalent dihedral angles for the two independent mol­ecules in the *E*-isomer are 19.37 (3) and 10.74 (6)° and 5.70 (11) and 9.74 (9)°, respectively (Hatayama & Okuno, 2012 ▸). In comparison with the *Z*-isomer, the *E*-isomer has a more planar conformation. The C=C bond length of the olefinic unit in the *Z*-isomer is *ca* 0.01 Å shorter than those of the *E*-isomer in spite of the steric repulsion. This is presumably because the reduced planarity of the *Z*-isomer decreases the contribution of the N^+^=C(H)—C(H)=B^−^ canonical structure.

In conclusion, structural analyses of both isomers of the hybrid π-system afford an important insight showing the discussed dihedral angles play a crucial role for contribution of the ionic canonical structure.

## Synthesis and crystallization

A solution of [Rh(cod)Cl]_2_ (0.024 g, 0.050 mmol), tri­cyclo­hexyl­phosphane (0.056 g, 0.198 mmol) and 4,4,5,5-tetra­methyl-1,3,2-dioxaborolane (0.478 ml, 3.30 mmol) in a mixture of cyclo­hexane (10 ml) and tri­ethyl­amine (2.3 ml, 17 mmol) was stirred for 3 h under an Ar atmosphere. Powdered 9-ethynyl-9*H*-carbazole (0.78 g, 4.1 mmol) was added to the solution followed by stirring for 4 h, also under Ar. After a filtration, the filtrate was concentrated under reduced pressure. The residue was extracted with CHCl_3_, and the solvent was removed *via* a rotary evaporator. The crude product was purified by gel permeation chromatography (GPC) (0.020 g, 1.5%). ^1^H NMR (400 MHz, CDCl_3_): δ 1.05 (*s*, 12H); 5.64 (*d*, *J* = 11.0 Hz, 1H); 7.25 (*t*, *J* = 7.2 Hz, 2H); 7.41 (*t*, *J* = 8.2 Hz, 2H); 7.45 (*d*, *J* = 11.0 Hz, 1H); 7.47 (*d*, *J* = 8.2 Hz, 2H); 8.04 (*d*, *J* = 7.7 Hz, 2H).

Single crystals of the title compound suitable for X-ray crystallographic analysis were prepared by recrystallization from its hexane solution.

## Refinement

Crystal data, data collection and structure refinement details are summarized in Table 1 ▸.

## Supplementary Material

Crystal structure: contains datablock(s) global, I. DOI: 10.1107/S2414314621001425/tk4068sup1.cif

Structure factors: contains datablock(s) I. DOI: 10.1107/S2414314621001425/tk4068Isup2.hkl

CCDC reference: 2061730

Additional supporting information:  crystallographic information; 3D view; checkCIF report

## Figures and Tables

**Table 1:** Experimental details

Crystal data	
Chemical formula	C_20_H_22_BNO_2_
*M*_r_	319.21
Crystal system, space group	Triclinic, *P* 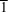
Temperature (K)	93
*a*, *b*, *c* (Å)	8.204 (3), 9.700 (5), 11.330 (5)
α, β, γ (°)	80.975 (15), 81.242 (17), 78.726 (17)
*V* (Å^3^)	866.4 (7)
*Z*	2
Radiation type	Mo *K*α
μ (mm^−1^)	0.08
Crystal size (mm)	0.20 × 0.10 × 0.04

Data collection	
Diffractometer	Rigaku Saturn724+
Absorption correction	Numerical (*NUMABS*; Rigaku, 1999 ▸)
*T*_min_, *T*_max_	0.990, 0.997
No. of measured, independent and observed [*F* ^2^ > 2.0σ(*F* ^2^)] reflections	5921, 3005, 2462
*R*_int_	0.041
(sin θ/λ)_max_ (Å^−1^)	0.594

Refinement	
*R*[*F* ^2^ > 2σ(*F* ^2^)], *wR*(*F* ^2^), *S*	0.054, 0.148, 1.04
No. of reflections	3005
No. of parameters	217
H-atom treatment	H-atom parameters not refined
Δρ_max_, Δρ_min_ (e Å^−3^)	0.24, −0.30

Computer programs: *CrystalClear* (Rigaku, 2008 ▸), *SHELXD* and *SHELXL* (Sheldrick, 2008 ▸), *OPTEP-3 for Windows* (Farrugia, 2012 ▸) and *publCIF* (Westrip, 2010 ▸).

## full crystallographic data

### Crystal data

**Table:**

C_20_H_22_BNO_2_	*Z* = 2
*M**_r_* = 319.21	*F*(000) = 340.00
Triclinic, *P*1	*D*_x_ = 1.224 Mg m^−^^3^
*a* = 8.204 (3) Å	Mo *K*α radiation, λ = 0.71075 Å
*b* = 9.700 (5) Å	Cell parameters from 2244 reflections
*c* = 11.330 (5) Å	θ = 1.8–25.0°
α = 80.975 (15)°	µ = 0.08 mm^−^^1^
β = 81.242 (17)°	*T* = 93 K
γ = 78.726 (17)°	Chip, colorless
*V* = 866.4 (7) Å^3^	0.20 × 0.10 × 0.04 mm

### Data collection

**Table:**

Rigaku Saturn724+ diffractometer	2462 reflections with *F*^2^ > 2.0σ(*F*^2^)
Detector resolution: 28.445 pixels mm^-1^	*R*_int_ = 0.041
ω scans	θ_max_ = 25.0°, θ_min_ = 3.0°
Absorption correction: numerical (NUMABS; Rigaku, 1999)	*h* = −9→9
*T*_min_ = 0.990, *T*_max_ = 0.997	*k* = −11→10
5921 measured reflections	*l* = −13→13
3005 independent reflections	

### Refinement

**Table:**

Refinement on *F*^2^	Secondary atom site location: difference Fourier map
*R*[*F*^2^ > 2σ(*F*^2^)] = 0.054	Hydrogen site location: inferred from neighbouring sites
*wR*(*F*^2^) = 0.148	H-atom parameters not refined
*S* = 1.04	*w* = 1/[σ^2^(*F*_o_^2^) + (0.0817*P*)^2^ + 0.3242*P*] where *P* = (*F*_o_^2^ + 2*F*_c_^2^)/3
3005 reflections	(Δ/σ)_max_ < 0.001
217 parameters	Δρ_max_ = 0.24 e Å^−^^3^
0 restraints	Δρ_min_ = −0.30 e Å^−^^3^
Primary atom site location: structure-invariant direct methods	

### Special details

**Table:**

Geometry. All esds (except the esd in the dihedral angle between two l.s. planes) are estimated using the full covariance matrix. The cell esds are taken into account individually in the estimation of esds in distances, angles and torsion angles; correlations between esds in cell parameters are only used when they are defined by crystal symmetry. An approximate (isotropic) treatment of cell esds is used for estimating esds involving l.s. planes.
Refinement. Refinement was performed using all reflections. The weighted R-factor (wR) and goodness of fit (S) are based on F^2^. R-factor (gt) are based on F. The threshold expression of F^2^ > 2.0 sigma(F^2^) is used only for calculating R-factor (gt).The C-bound H atoms were placed at ideal positions and were refined as riding on their parent C atoms. *U*_iso_(H) values of the H atoms were set at 1.2*U*_eq_(parent atom for C_sp2_) and 1.5 *U*_eq_(parent atom for C_sp3_).

### Fractional atomic coordinates and isotropic or equivalent isotropic displacement parameters (Å^2^)

**Table:**

	*x*	*y*	*z*	*U*_iso_*/*U*_eq_	
O1	0.20319 (15)	0.34856 (14)	1.00915 (12)	0.0230 (3)	
O2	0.38376 (15)	0.17377 (14)	1.10767 (12)	0.0239 (3)	
N1	0.41036 (19)	0.40247 (16)	0.74648 (14)	0.0205 (4)	
C1	0.3703 (2)	0.5164 (2)	0.65803 (17)	0.0216 (4)	
C2	0.4130 (2)	0.6510 (2)	0.64047 (19)	0.0270 (5)	
C3	0.3585 (3)	0.7445 (2)	0.5436 (2)	0.0326 (5)	
C4	0.2609 (3)	0.7075 (2)	0.46672 (19)	0.0323 (5)	
C5	0.2164 (2)	0.5748 (2)	0.48530 (18)	0.0281 (5)	
C6	0.2716 (2)	0.4769 (2)	0.58157 (17)	0.0216 (4)	
C7	0.2548 (2)	0.3314 (2)	0.62426 (16)	0.0204 (4)	
C8	0.1799 (2)	0.2338 (2)	0.58267 (18)	0.0252 (5)	
C9	0.1991 (2)	0.0959 (2)	0.63799 (19)	0.0272 (5)	
C10	0.2929 (2)	0.0528 (2)	0.73549 (19)	0.0267 (5)	
C11	0.3653 (2)	0.1483 (2)	0.78009 (17)	0.0223 (4)	
C12	0.3436 (2)	0.28763 (19)	0.72443 (16)	0.0190 (4)	
C13	0.5305 (2)	0.3982 (2)	0.82481 (18)	0.0236 (4)	
C14	0.5195 (2)	0.3395 (2)	0.93873 (17)	0.0228 (4)	
C15	0.1022 (2)	0.2904 (2)	1.11685 (17)	0.0222 (4)	
C16	0.2149 (2)	0.1462 (2)	1.15663 (18)	0.0226 (4)	
C17	0.0748 (3)	0.3957 (2)	1.2068 (2)	0.0312 (5)	
C18	−0.0659 (2)	0.2788 (2)	1.0809 (2)	0.0291 (5)	
C19	0.2125 (3)	0.1056 (2)	1.29157 (19)	0.0326 (5)	
C20	0.1851 (2)	0.0232 (2)	1.09897 (19)	0.0285 (5)	
B1	0.3668 (3)	0.2849 (2)	1.0180 (2)	0.0212 (5)	
H2	0.4774	0.6774	0.6934	0.032*	
H3	0.3882	0.8361	0.5289	0.039*	
H4	0.2249	0.7744	0.4010	0.039*	
H5	0.1489	0.5503	0.4333	0.034*	
H8	0.1165	0.2624	0.5168	0.030*	
H9	0.1486	0.0291	0.6100	0.033*	
H10	0.3067	−0.0434	0.7715	0.032*	
H11	0.4278	0.1194	0.8465	0.027*	
H13	0.6280	0.4470	0.7822	0.028*	
H14	0.6200	0.3320	0.9790	0.037*	
H17A	0.0075	0.3607	1.2804	0.037*	
H17B	0.1834	0.4073	1.2262	0.037*	
H17C	0.0159	0.4874	1.1718	0.037*	
H18A	−0.1358	0.2401	1.1515	0.035*	
H18B	−0.1225	0.3730	1.0494	0.035*	
H18C	−0.0474	0.2158	1.0186	0.035*	
H19A	0.1013	0.0863	1.3272	0.039*	
H19B	0.2966	0.0203	1.3079	0.039*	
H19C	0.2382	0.1835	1.3270	0.039*	
H20A	0.0733	0.0020	1.1298	0.034*	
H20B	0.1935	0.0488	1.0113	0.034*	
H20C	0.2697	−0.0606	1.1186	0.034*	

### Atomic displacement parameters (Å^2^)

**Table:**

	*U*^11^	*U*^22^	*U*^33^	*U*^12^	*U*^13^	*U*^23^
O1	0.0134 (7)	0.0253 (7)	0.0279 (8)	−0.0019 (5)	−0.0006 (5)	−0.0002 (6)
O2	0.0119 (7)	0.0310 (8)	0.0269 (8)	−0.0027 (5)	−0.0019 (5)	0.0002 (6)
N1	0.0150 (8)	0.0221 (9)	0.0245 (8)	−0.0050 (6)	−0.0014 (6)	−0.0022 (7)
C1	0.0137 (9)	0.0220 (10)	0.0256 (10)	−0.0010 (7)	0.0058 (7)	−0.0031 (8)
C2	0.0174 (10)	0.0248 (10)	0.0360 (12)	−0.0037 (8)	0.0065 (8)	−0.0052 (9)
C3	0.0241 (11)	0.0224 (11)	0.0421 (13)	0.0013 (8)	0.0108 (9)	0.0018 (9)
C4	0.0258 (11)	0.0287 (12)	0.0323 (12)	0.0058 (9)	0.0062 (9)	0.0035 (9)
C5	0.0209 (10)	0.0296 (11)	0.0270 (11)	0.0056 (8)	0.0032 (8)	−0.0019 (9)
C6	0.0144 (9)	0.0243 (10)	0.0223 (10)	0.0008 (7)	0.0048 (7)	−0.0041 (8)
C7	0.0114 (9)	0.0261 (10)	0.0214 (10)	0.0008 (7)	0.0022 (7)	−0.0058 (8)
C8	0.0157 (9)	0.0326 (11)	0.0277 (11)	−0.0001 (8)	−0.0023 (8)	−0.0105 (9)
C9	0.0185 (10)	0.0308 (11)	0.0362 (12)	−0.0062 (8)	−0.0028 (8)	−0.0141 (9)
C10	0.0204 (10)	0.0236 (10)	0.0343 (11)	−0.0036 (8)	0.0020 (8)	−0.0043 (8)
C11	0.0158 (9)	0.0252 (10)	0.0250 (10)	−0.0038 (8)	0.0010 (8)	−0.0032 (8)
C12	0.0120 (9)	0.0222 (10)	0.0222 (10)	−0.0038 (7)	0.0028 (7)	−0.0048 (7)
C13	0.0148 (9)	0.0257 (10)	0.0324 (11)	−0.0078 (8)	−0.0012 (8)	−0.0065 (8)
C14	0.0135 (9)	0.0266 (10)	0.0298 (11)	−0.0033 (8)	−0.0035 (8)	−0.0077 (8)
C15	0.0132 (9)	0.0257 (10)	0.0269 (10)	−0.0043 (7)	0.0002 (7)	−0.0024 (8)
C16	0.0131 (9)	0.0272 (10)	0.0265 (10)	−0.0045 (8)	−0.0002 (7)	−0.0013 (8)
C17	0.0217 (11)	0.0338 (12)	0.0388 (12)	−0.0069 (9)	0.0044 (9)	−0.0125 (10)
C18	0.0145 (10)	0.0297 (11)	0.0433 (13)	−0.0047 (8)	−0.0055 (8)	−0.0027 (9)
C19	0.0261 (11)	0.0415 (13)	0.0278 (11)	−0.0075 (9)	−0.0012 (9)	0.0026 (9)
C20	0.0217 (10)	0.0260 (11)	0.0360 (12)	−0.0038 (8)	−0.0003 (9)	−0.0023 (9)
B1	0.0150 (10)	0.0245 (11)	0.0249 (11)	−0.0018 (8)	−0.0027 (8)	−0.0073 (9)

### Geometric parameters (Å, º)

**Table:**

O1—B1	1.375 (2)	C10—H10	0.9505
O1—C15	1.467 (2)	C11—C12	1.389 (3)
O2—B1	1.362 (3)	C11—H11	0.9501
O2—C16	1.471 (2)	C13—C14	1.324 (3)
N1—C1	1.395 (3)	C13—H13	1.0277
N1—C12	1.404 (3)	C14—B1	1.557 (3)
N1—C13	1.414 (3)	C14—H14	0.9869
C1—C2	1.393 (3)	C15—C17	1.517 (3)
C1—C6	1.412 (3)	C15—C18	1.525 (3)
C2—C3	1.380 (3)	C15—C16	1.561 (3)
C2—H2	0.9501	C16—C19	1.516 (3)
C3—C4	1.396 (3)	C16—C20	1.522 (3)
C3—H3	0.9502	C17—H17A	0.9797
C4—C5	1.383 (3)	C17—H17B	0.9805
C4—H4	0.9503	C17—H17C	0.9804
C5—C6	1.398 (3)	C18—H18A	0.9798
C5—H5	0.9509	C18—H18B	0.9799
C6—C7	1.445 (3)	C18—H18C	0.9805
C7—C8	1.397 (3)	C19—H19A	0.9804
C7—C12	1.407 (3)	C19—H19B	0.9808
C8—C9	1.375 (3)	C19—H19C	0.9800
C8—H8	0.9496	C20—H20A	0.9798
C9—C10	1.405 (3)	C20—H20B	0.9805
C9—H9	0.9497	C20—H20C	0.9807
C10—C11	1.386 (3)		
			
B1—O1—C15	106.68 (15)	N1—C13—H13	111.4
B1—O2—C16	107.55 (14)	C13—C14—B1	128.25 (18)
C1—N1—C12	108.46 (16)	C13—C14—H14	115.7
C1—N1—C13	123.14 (16)	B1—C14—H14	116.1
C12—N1—C13	126.85 (16)	O1—C15—C17	106.93 (15)
C2—C1—N1	129.49 (19)	O1—C15—C18	107.93 (16)
C2—C1—C6	121.62 (18)	C17—C15—C18	109.52 (16)
N1—C1—C6	108.89 (17)	O1—C15—C16	102.76 (14)
C3—C2—C1	117.8 (2)	C17—C15—C16	113.79 (17)
C3—C2—H2	121.1	C18—C15—C16	115.22 (16)
C1—C2—H2	121.1	O2—C16—C19	107.36 (15)
C2—C3—C4	121.6 (2)	O2—C16—C20	107.45 (15)
C2—C3—H3	119.2	C19—C16—C20	110.32 (17)
C4—C3—H3	119.3	O2—C16—C15	102.16 (14)
C5—C4—C3	120.7 (2)	C19—C16—C15	115.19 (17)
C5—C4—H4	119.7	C20—C16—C15	113.57 (17)
C3—C4—H4	119.6	C15—C17—H17A	109.5
C4—C5—C6	119.2 (2)	C15—C17—H17B	109.4
C4—C5—H5	120.5	H17A—C17—H17B	109.5
C6—C5—H5	120.4	C15—C17—H17C	109.4
C5—C6—C1	119.20 (18)	H17A—C17—H17C	109.5
C5—C6—C7	134.00 (19)	H17B—C17—H17C	109.4
C1—C6—C7	106.78 (16)	C15—C18—H18A	109.5
C8—C7—C12	119.23 (18)	C15—C18—H18B	109.5
C8—C7—C6	133.36 (18)	H18A—C18—H18B	109.5
C12—C7—C6	107.30 (16)	C15—C18—H18C	109.5
C9—C8—C7	119.36 (18)	H18A—C18—H18C	109.4
C9—C8—H8	120.4	H18B—C18—H18C	109.4
C7—C8—H8	120.3	C16—C19—H19A	109.5
C8—C9—C10	120.67 (18)	C16—C19—H19B	109.5
C8—C9—H9	119.7	H19A—C19—H19B	109.4
C10—C9—H9	119.6	C16—C19—H19C	109.5
C11—C10—C9	121.08 (19)	H19A—C19—H19C	109.5
C11—C10—H10	119.5	H19B—C19—H19C	109.4
C9—C10—H10	119.5	C16—C20—H20A	109.6
C10—C11—C12	117.78 (18)	C16—C20—H20B	109.5
C10—C11—H11	121.1	H20A—C20—H20B	109.5
C12—C11—H11	121.1	C16—C20—H20C	109.5
C11—C12—N1	129.49 (18)	H20A—C20—H20C	109.4
C11—C12—C7	121.82 (18)	H20B—C20—H20C	109.4
N1—C12—C7	108.51 (16)	O2—B1—O1	113.61 (17)
C14—C13—N1	124.70 (17)	O2—B1—C14	122.75 (17)
C14—C13—H13	123.9	O1—B1—C14	123.54 (18)
			
C12—N1—C1—C2	−178.03 (18)	C13—N1—C12—C7	−168.45 (16)
C13—N1—C1—C2	−11.4 (3)	C8—C7—C12—C11	2.8 (3)
C12—N1—C1—C6	2.38 (19)	C6—C7—C12—C11	−173.95 (16)
C13—N1—C1—C6	169.01 (16)	C8—C7—C12—N1	178.38 (16)
N1—C1—C2—C3	179.05 (17)	C6—C7—C12—N1	1.58 (19)
C6—C1—C2—C3	−1.4 (3)	C1—N1—C13—C14	144.8 (2)
C1—C2—C3—C4	1.3 (3)	C12—N1—C13—C14	−51.1 (3)
C2—C3—C4—C5	−0.3 (3)	N1—C13—C14—B1	−10.5 (3)
C3—C4—C5—C6	−0.6 (3)	B1—O1—C15—C17	97.21 (18)
C4—C5—C6—C1	0.5 (3)	B1—O1—C15—C18	−145.05 (16)
C4—C5—C6—C7	−177.45 (19)	B1—O1—C15—C16	−22.88 (19)
C2—C1—C6—C5	0.5 (3)	B1—O2—C16—C19	−142.55 (17)
N1—C1—C6—C5	−179.86 (15)	B1—O2—C16—C20	98.79 (18)
C2—C1—C6—C7	178.99 (16)	B1—O2—C16—C15	−20.98 (19)
N1—C1—C6—C7	−1.38 (19)	O1—C15—C16—O2	26.31 (18)
C5—C6—C7—C8	1.9 (4)	C17—C15—C16—O2	−88.90 (18)
C1—C6—C7—C8	−176.28 (19)	C18—C15—C16—O2	143.41 (16)
C5—C6—C7—C12	178.03 (19)	O1—C15—C16—C19	142.33 (16)
C1—C6—C7—C12	−0.13 (19)	C17—C15—C16—C19	27.1 (2)
C12—C7—C8—C9	−2.1 (3)	C18—C15—C16—C19	−100.6 (2)
C6—C7—C8—C9	173.73 (18)	O1—C15—C16—C20	−89.07 (18)
C7—C8—C9—C10	0.0 (3)	C17—C15—C16—C20	155.71 (17)
C8—C9—C10—C11	1.4 (3)	C18—C15—C16—C20	28.0 (2)
C9—C10—C11—C12	−0.6 (3)	C16—O2—B1—O1	7.5 (2)
C10—C11—C12—N1	−175.98 (18)	C16—O2—B1—C14	−176.06 (17)
C10—C11—C12—C7	−1.5 (3)	C15—O1—B1—O2	10.7 (2)
C1—N1—C12—C11	172.61 (18)	C15—O1—B1—C14	−165.67 (17)
C13—N1—C12—C11	6.6 (3)	C13—C14—B1—O2	148.6 (2)
C1—N1—C12—C7	−2.46 (19)	C13—C14—B1—O1	−35.3 (3)
